# Cancer Diagnosis Disclosure and Quality of Life in Elderly Cancer Patients

**DOI:** 10.3390/healthcare7040163

**Published:** 2019-12-14

**Authors:** Ying Zheng, Fang Lei, Bao Liu

**Affiliations:** 1 School of Public Health, Fudan University, Shanghai 200032, China; zhengy16@fudan.edu.cn (Y.Z.); liub@fudan.edu.cn (B.L.); 2 School of Nursing, University of California, Los Angeles, CA 90095, USA

**Keywords:** cancer diagnosis disclosure, cancer patients, caregiver, inform, SPIKES, Medical Coping Modes Questionnaire

## Abstract

Informed consent and patient autonomy rights require an optimal cancer diagnosis disclosure strategy to be used to update the patients and caregivers with the bad news. However, a cancer diagnosis disclosure may arouse anxiety and distress which increase patients’ and caregivers’ psychological burden. This study aims to explore the influence of cancer diagnosis disclosure on the quality of life in elderly cancer patients and their caregivers, and to introduce an effective way to disclose cancer diagnosis. A total of 120 participants were randomly selected in the study. The Medical Coping Modes Questionnaire was used to select informed person. The SPIKES was used to guide the disclosure procedures. The informed patients’ or caregivers’ quality of life were evaluated by the Generic Quality of Life Inventory-74 or Caregiver Quality of Life Scale before and after the cancer diagnosis disclosure and at the discharge time. After cancer diagnosis disclosure, no significant change was found on the quality of life of the informed person. With multiple psychological interventions implemented, a significant increase was noticed on the quality of life of the informed person. Applying the cancer diagnosis disclosure strategies and psychological intervention were essential to improve cancer patients’ and caregivers’ quality of life.

## 1. Introduction

Informed consent is a legal and ethical concept. It refers to a medical permission or decision given by a patient after being informed about the diagnosis, treatment, tests, or procedures related to a disease [1]. Also, patient autonomy rights empowers patients to choose proper treatment and make medical decisions based on their medical diagnosis [2,3]. However, knowing a life-threatening diagnosis (e.g., cancer diagnosis) often imposes a crisis on the patients. They have to confront the illness and its treatment. They also have to deal with issues related to the meaning of life, death, an uncertain future, and negative emotions (e.g., anxiety, distress, depression) related to the cancer diagnosis [4], which may decrease their quality of life and shorten the prognosis.

In China, the most common approach to disclose a cancer diagnosis is the “family consent for disclosure” method [4]. According to the law of the People’s Republic of China on Medical Practitioners, health care providers have the obligation to disclose the truth to the patients and their family members; they also have the responsibility to take necessary actions to avoid adverse consequences related to the diagnosis disclosure [5]. Therefore, in clinical practice in China, the “family consent for disclosure” approach was frequently used by the health care providers to disclose a life-threatening diagnosis (e.g., cancer diagnosis) [6]. In these circumstances, health care providers choose to disclose patients’ cancer diagnosis to their caregivers primarily, and then caregivers make the decision about further cancer diagnosis disclosure [6,7]. This diagnosis disclosure strategy avoids a direct interaction between health care providers and patients. It reduces the possibility of bringing a high level of anxiety and distress to patients. However, from the legal and ethical aspects, it is inconsistent with patients’ informed consent and personal autonomy rights [1]. Furthermore, in this situation, caregivers were placed in a difficult dilemma. They had to decide whether to disclose the cancer diagnosis to the patient, how, when, and what to do to reduce unnecessary anxiety and distress brought to the patient. It may increase the anxiety and distress level of caregivers, thus affecting the quality of supportive care they provided to the patients.

According to Leckie [8], appropriate information could help patients know their health condition, improve their participation in the treatment, decrease their anxiety and distress levels, help them to feel safe, satisfied, and valued, and meet the psychological needs of patients with self-esteem and respect [8]. As a result, patients’ psychological function and social function improved significantly. Also, results from the study conducted by Montgomery et al. [9] showed that due to the undisclosed diagnosis, patients may not be able to fully understand and adhere to the treatment. With the progress of the treatment and the occurrence of adverse reactions, patients may suspect the kindly-told diagnosis or undisclosed diagnosis. When there was no way to confirm, patients’ anxiety or distress levels were even higher than that of patients who had already known their disease diagnosis [9]. Therefore, cancer diagnosis disclosure should be done with caution and well preparedness.

The SPIKES protocol is a ‘bad news’ delivering guideline which is popularly used in the United States [10]. It has reached guideline status in America and in a number of other countries [11]. It is used as a guide for sensitive practice and for communication skills training [12], with a special application for cancer patients [13]. However, there is a lack of reports about its application in Chinese health care settings.

With the purpose of exploring the influence of cancer diagnosis disclosure and psychological care intervention on the quality of life of the informed person (patients or caregivers), this study applied the SPIKES protocol to disclose cancer diagnosis in elderly cancer patients and implemented psychological care for the informed person after cancer diagnosis disclosure. The informed person’s quality of life was measured sequentially to test the effect of cancer diagnosis disclosure and psychological care intervention. The hypothesis related to the aims of the study were: 1. No significant decrease was found in the informed person’s quality of life after the cancer diagnosis was disclosed; 2. The informed person’s quality of life increased significantly after the psychological care intervention was implemented.

## 2. Materials and Methods

### 2.1. Sample

The participants were randomly chosen from the Inpatient Center of a top-ten hospital in China between June 2012 and June 2014. Inclusion criteria for the patients participating in the study were: 1. First time diagnosed with cancer by pathology or cytology criteria; 2. Had not been informed of the newly diagnosed cancer diagnosis; 3. Had no other serious diseases; 4. Aged 60 years or older; 5. Had no mental illness history; 6. Were conscious and able to volunteer to participate in the study; and 7. Their remaining life expectancy was more than 3 months. The patients were recruited by physicians’ referral and each patient identified a person as the caregiver. Initially, 127 newly diagnosed elderly cancer patients and their caregivers participated in the study. However, seven dyads withdrew from the study because of either loss to follow-up (*n* = 3) or refused participation (*n* = 4).

### 2.2. Instruments

#### 2.2.1. Medical Coping Modes Questionnaire

The Chinese version Medical Coping Modes Questionnaire was translated and adapted by Qianjing Jiang [14]. It was used to evaluate patients’ and caregivers’ coping styles [15,16]. The questionnaire included three categories: Face, avoid, and surrender, with a total of 20 items. Each item was measured by a Likert score ranging from 1 to 4. Eight of the 20 items were reverse scores. In this study, the Cronbach’s coefficient of the three categories were 0.89, 0.80, and 0.86; retest reliabilities were 0.86, 0.85, and 0.89.

#### 2.2.2. Generic Quality of Life Inventory-74

The patients’ quality of life was evaluated using Generic Quality of Life Inventory-74 (GQOLI-74), which was a self-rating Chinese scale used to evaluate cancer patients’ quality of life [17]. It had 70 items measuring four functions of patients’ quality of life: Physical function (20 items), psychological function (20 items), social function (20 items), and physical life (10 items). Four extra items were used to evaluate the overall quality of life. Each item was measured by a Likert score ranging from 1 to 5. In this study, the Cronbach’s a coefficient of the four dimensions were 0.87, 0.89, 0.83, and 0.85; retest reliabilities were 0.86, 0.87, 0.81, and 0.84. Total scores ranged from 0 to 350 points. All scores were positively related to the quality of life, which meant the higher the scores, the better the quality of life.

#### 2.2.3. Caregiver Quality of Life Scale

The caregivers’ quality of life was evaluated using Chinese Caregiver Quality of Life (CQOL) Scale [18,19], which was developed and provided by BECKMAN National Medical Research Center, and then translated and validated by other Chinese researchers [18]. It had 37 items measuring four dimensions: Physical function, psychological function, social function, and spiritual life. This study removed two items about religion in the spiritual life dimension because they were not applicable to this study. In this study, the Cronbach’s coefficient of the four dimensions in the modified scale were 0.85, 0.88, 0.89, and 0.81; retest reliabilities were 0.86, 0.85, 0.74, and 0.89. Total scores of the modified scale ranged from 0 to 350 points. A higher score indicated a higher quality of life.

### 2.3. Intervention

The specific cancer diagnosis disclosure procedure was implemented around three core elements: Selecting the informed person, cancer diagnosis disclosure, and psychological care after the cancer diagnosis disclosure.

#### 2.3.1. Selecting the Informed Person

In this study, the Medical Coping Modes Questionnaire was used for selecting the cancer diagnosis informed person. The patients’ and their caregivers’ coping styles were tested by answering the Medical Coping Modes Questionnaire when the patients admitted to the hospital. The patient and the caregiver’s scores on each coping category were compared. Finally, the person (patient or the caregiver) whose average facing score was much higher than the average reverse scores (avoid and surrender) was selected to be disclosed with the cancer diagnosis. At last, 48 patients and 72 caregivers were included in the study as the informed person. They were asked to fill the quality of life scale at three points of time: One day before and one day after the diagnosis were disclosed, and before the discharge time.

#### 2.3.2. Cancer Diagnosis Disclosure Steps

The cancer diagnosis disclosure procedure was implemented following the SPIKES steps [20,21,22,23]. The six steps of SPIKES included setting up the interview (S), assessing the patient’s perception (P), obtaining the patient’s invitation (I), giving knowledge and information to the patient (K), addressing the patient’s emotions with empathic responses (E), and strategy and summary (S). Following the SPIKES six-step strategy [23], the health care team (physician, nurse manager, and charge nurse) arranged a private face to face meeting in a separate room with the informed person, and the most trusted person of the informed person was present, such as close friends or other caregivers. Before the cancer diagnosis disclosure procedure, a heuristic conversation was induced to evaluate the informed person’s psychological condition and the knowledge level of the cancer, to prepare the informed person with a better understanding about the disease and help the person to prepare for a possible emotion fluctuation. The health care team disclosed the cancer diagnosis gradually, avoiding too many professional words. Different amounts of information were provided according to the informed person’s knowledge level, information demand level, and the prognosis of the cancer. Basic information disclosure content included the patient’s present stage of cancer, alternative treatment options and risks, prognosis, and medical costs.

#### 2.3.3. Psychological Care after the Diagnosis Disclosure

After the cancer diagnosis was disclosed, the informed person’s psychological reaction was observed carefully. Understanding and comfort were expressed until the informed person calmed down. A plan for future life and treatment was made together. Furthermore, tailored supportive psychological nursing care (e.g., psychological consultation) was provided by the psychological experts weekly. The informed person was also introduced to the group of people who had—or whose family member had—the same diagnosis. A variety of activities (e.g., group discussions, lectures, ‘life experience-sharing’ tea meetings, ‘road to anti-cancer’ consultation) were organized weekly by the group leaders (two charge nurses) to help the informed person to increase self-efficacy to deal with cancer. ‘Talking treatment’ service and spiritual support were provided by charge nurses biweekly (Table 1). Questions raised by the informed person were answered promptly by the health care team. Close contact was kept between the health care team and the informed person.

### 2.4. Statistical Analysis

Data was analyzed using SPSS 24.0 software (SPSS Inc., Chicago, IL, USA). Descriptive analysis and repeated measures ANOVA were used to compare the quality of life of the informed person at three different time points. The *p* value was set at the 0.05 level.

## 3. Results

### 3.1. Sample Characteristics

Among the 120 participants, 72 participants (60%) were male and 48 were female. Seventy-seven participants (64.2%) were 60 to 70 years old, and 43 participants were over 70 years old. The mean age of the participants was 68.3 years old. For the cancer diagnosis, 35 were lung cancer, 21 were liver cancer, 16 were breast cancer, 15 were gastric cancer, nine were colon cancer, and 24 were other cancers. The education levels of the participants were as follows: 45 were college level and above, 62 were high school level, 10 were middle school level, three were elementary school level. Sixty-four of the participants (53.3%) were currently married, and 56 of the participants were divorced, widowed, or single (Table 2).

### 3.2. Changes in the Psychological Function and Social Function

The results showed that although the average psychological function and social function scores decreased, no significant difference was found after the SPIKES strategy was applied to disclose the cancer diagnosis (*p* > 0.05). However, after the psychological care intervention, the psychological function and social function scores of the informed person increased significantly at discharge time (8.23 ± 4.37), which were significantly higher than the scores one day after the patients’ diagnoses were disclosed (*p* < 0.05) (Table 3 and Table 4).

### 3.3. Changes in the Physical Function and Physical/Spiritual Life

As the results in Table 3 and Table 4 showed, although the average physical function and physical/spiritual life scores decreased, similarly, no significant decrease was found after the SPIKES strategy was applied to disclose the cancer diagnosis (*p* > 0.05). Also, after the psychological care intervention, there were no significant decrease in the physical function and physical/spiritual life scores at discharge time (*p* > 0.05).

### 3.4. Changes in the Quality of Life

As the results showed, although the average quality of life scores decreased, similarly, no significant difference was found after the SPIKES strategy was applied to disclose the cancer diagnosis (*p* > 0.05). However, after the psychological care intervention, there were significant increases in the quality of life scores both in patients and caregivers at discharge time (*p* < 0.05) (Table 3 and Table 4).

## 4. Discussion

To our knowledge, to date, this study is the first application study using SPIKES protocol to disclose cancer diagnosis among Chinese patients. Cancer diagnosis disclosure is a difficult situation to be handled, which may arouse long-term distress and anxiety [24,25,26]. Previous studies [27,28,29] showed knowing a cancer diagnosis had a significant influence on the informed person’s psychological and social functions. However, in this study, we used the Medical Coping Modes Questionnaire to select the informed person, which potentially decreased negative effects on the psychological vulnerable informed person. Also, we used the SPIKES strategy to disclose the cancer diagnosis, which helped to avoid a significant change on the informed person’s psychological and social functions. Although in the early stage after the cancer diagnosis was disclosed, most of the informed persons were not able to accept the reality right away, however, with a carefully preparation instructed by the SPIKES strategy, we avoided bringing unnecessary distress and anxiety to the informed person during the cancer diagnosis disclosure. We helped the informed person to overcome the stages of the negative psychological emotions (e.g., “tumor psychological shock period”, which was characterized by obvious anxiety, fear, and other negative emotions [30]; the stage of defense and denial which was characterized by fear of death, being scared of radiation, chemotherapy, and surgery, and worrying about medical costs [31]; and the stage of reluctant acceptance, which was characterized by the acceptance of their roles as cancer patients or the caregivers of cancer patients [32,33]) with a positive attitude, thus the informed person was able to transit through the stages of negative psychological emotion within a short period.

Furthermore, the tailored psychological interventions improved the psychological and social function of the informed person, thus increasing the quality of life of the informed person. After taking multiple psychological interventions, such as giving pertinent and tailored psychological counseling, the informed person got out from the “tumor psychological shock period” promptly and developed trust in the health care providers. Also, connecting the informed person with the cancer group helped them to develop confidence to fight with cancer. The group activities which launched on a regular basis also offered them an opportunity to exchange their experiences and provided them a chance to share and solve their confusions toward cancer treatments. Furthermore, they provided ‘talking treatment’ service and spiritual support helped the informed person to form a positive belief towards death. It also provided us with valuable opportunities to be aware of and prevent multiple predictable accidents (e.g., suicides).

Due to the limitation of time and resources, this study had several limitations. First, the sample was randomized from one chosen hospital in China, which may limit the result to be generalized to other settings. Second, this study was a quasi-experimental study which lacked a control group. This may not be able to eliminate the effect of confounders. Lastly, some of the caregivers were chosen to be informed about the cancer diagnosis in this study. This required more work when the diagnosis needed to be further disclosed to the patients, which may increase the workload of the health care team.

## 5. Conclusions

Cancer diagnosis disclosure is tough, which may arouse distress and anxiety to the informed person. However, with the appropriate disclosure strategy, we avoided a significant decrease in the informed person’s quality of life. After implementing multiple psychological care intervention, we helped the informed person increase their quality of life. As a vulnerable population, cancer patients and caregivers should be updated by their cancer diagnosis with an optimal method. More research should be done to address the cancer diagnosis disclosure difficulty. Diverse psychological care routines should also be developed to help them overcome the trauma brought by cancer diagnosis disclosure.

## Figures and Tables

**Table 1:** Intervention Timeline.

Timeline	Intervention
At admission	Coping skill evaluation
One day before diagnosis disclosure	Quality of life evaluation
One day after diagnosis disclosure	Quality of life evaluation
Two day after diagnosis disclosure to one day before discharge	Weekly psychological consultation, group activity Biweekly ‘talking treatment’ service and spiritual support
At discharge	Quality of life evaluation

**Table 2:** Sample Characteristics (*N* = 120).

Item	Categories	*N*	Percentage
Gender	Male	72	60%
	Female	48	40%
Age	60–70 y	77	64.2%
	70 y above	43	35.8%
Diagnosis	Lung cancer	35	29.2%
	Liver cancer	21	17.5%
	Breast cancer	16	13.3%
	Gastric cancer	15	12.5%
	Colony cancer	9	7.5%
	Other cancers	24	20%
Education level	College and above	45	37.5%
	High school	62	51.7%
	Middle school	10	8.3%
	Elementary school	3	2.5%
Marital status	Married	64	53.3%
	Divorced, widowed or single	56	46.7%

**Table 3:** Quality of life of the patients (*n* = 48), X ± s.

Time	Before Disclosure	After Disclosure	*p* Value	Discharge	*p* Value ^1^
Physical Function	61.32 ± 5.7	61.26 ± 4.6	0.069	61.40 ± 4.3	0.078
Psychological Function	60.33 ± 3.3	59.72 ± 3.1	0.060	68.56 ± 5.5	0.044
Social Function	60.36 ± 5.5	59.21 ± 4.7	0.074	70.23 ± 4.2	0.037
Physical Life	32.53 ± 5.1	31.37 ± 5.3	0.067	33.43 ± 3.5	0.075
Total Score	213.74 ± 5.2	210.37 ± 4.2	0.065	219.77 ± 5.4	0.049

^1^ Discharge vs. After disclosure.

**Table 4:** Quality of life of the caregivers (*n* = 72), X ± s.

Time	Before Disclosure	After Disclosure	*p* Value	Discharge	*p* Value ^1^
Physical Function	63.37 ± 5.9	62.46 ± 4.8	0.083	64.34 ± 5.7	0.073
Psychological Function	61.38 ± 3.7	59.78 ± 3.6	0.065	70.51 ± 5.0	0.049
Social Function	63.36 ± 5.1	62.23 ± 4.1	0.069	72.23 ± 4.0	0.045
Spiritual Life	33.51 ± 5.07	32.31 ± 5.39	0.088	33.52 ± 3.57	0.097
Total Score	224.9 ± 5.3	223.9 ± 4.2	0.074	226.3 ± 4.7	0.047

^1^ Discharge vs. After disclosure.
